# PBMC transcriptomic signatures reflect *Trypanosoma cruzi* strain diversity and trained immunity in chronically infected macaques

**DOI:** 10.1172/jci.insight.186003

**Published:** 2025-01-07

**Authors:** Hans Desale, Weihong Tu, Kelly Goff, Preston A. Marx, Claudia Herrera, Eric Dumonteil

**Affiliations:** 1 Department of Tropical Medicine and Infectious Disease, School of Public Health and Tropical Medicine, and; 2 Vector-Borne and Infectious Disease Research Center, Tulane University, New Orleans, Louisiana, USA.; 3 Division of Microbiology, Tulane National Primate Research Center, Tulane University, Covington, Louisiana, USA.

**Keywords:** Infectious disease, Microbiology, Parasitology

## Abstract

Chagas disease is a tropical disease caused by *Trypanosoma cruzi* with clinical presentations ranging from asymptomatic to cardiac and/or gastrointestinal complications. The mechanisms of pathogenesis are still poorly understood, but *T*. *cruzi* strain diversity may be associated with disease progression. Therefore, we evaluated the transcriptomic response of PBMCs from macaques with natural chronic infections and tested for heterogeneity in their gene signatures. Remarkably, transcriptomic response to *T*. *cruzi* infection matched parasite strain profiles, indicating that parasite diversity is a key determinant of host response. While differences in adaptive immune responses were identified, more striking alterations of innate immune processes were detected. Thus, initial innate response to *T*. *cruzi* infection may be conditioned by parasite strain diversity, resulting in different profiles of trained immunity modulating subsequent adaptive responses, allowing parasite control or its persistence during the chronic phase. These results call for further characterization of the cross-talk between innate and adaptive immunity according to parasite diversity as well as how altered trained immunity contributes to pathogenesis, as this may lead to better treatments and vaccines.

## Abstract 2

Transcriptomic response to T. cruzi infection matched parasite strain profiles and suggested different profiles of trained immunity in naturally infected macaques

## Introduction

Chagas disease is a tropical disease with an estimated annual burden of $627.46 million in health-care costs and 806,170 disability-adjusted life years (DALYs) worldwide (1), caused by infections with the protozoan parasite *Trypanosoma cruzi*. Following infection, infected hosts undergo an acute phase that lasts a few weeks, characterized by flu-like nonspecific signs and symptoms and high levels of circulating blood parasites. Patients then enter the chronic phase, which is initially asymptomatic and often lasts decades. About 20%–40% will then progress to a symptomatic chronic phase with cardiac (e.g., cardiomyopathy) and/or gastrointestinal (e.g., megaesophagus and megacolon) complications of growing severity, leading to disability and death (2, 3). While these clinical manifestations are well described, the mechanisms of pathogenesis and disease progression are less understood, which impedes any reliable prognosis for patients. Indeed, there are no biomarkers that identify patients who will remain asymptomatic or those who will progress to cardiac or digestive disease (4), and this complicates patient care.

The immune response to *T*. *cruzi* infection has been extensively studied to understand the ability of parasites to evade host immune responses and persist over prolonged time, and to identify immune mechanisms allowing parasite control. Current understanding suggests that the initial steps of *T*. *cruzi* infection and replication occur inside resident monocytes/macrophages at the parasite entry site, leading to the activation of innate mechanisms including type I IFN signaling and the recruitment of neutrophils and NK cells, but these cells fail to be fully activated, resulting in a delayed immune response favoring parasite establishment (5–7). A robust and effective B and T cell response is eventually induced, allowing for parasite control and the end of the acute phase, but the head start of the parasite is such that sterilizing immunity cannot be reached and low levels of parasite can persist for a prolonged time (8–10).

During the chronic phase, adaptive immunity is considered the most important mechanism contributing to parasite control through antigen-specific cytolytic T cells and antibody production by B cells as well as a balanced Th1/Th2/Th17 response (8, 11). Thus, a hyperpolarized Th1 response may lead to exacerbated inflammation and tissue damage, while immune exhaustion or a Th2 response may allow for parasite proliferation, both allowing for disease progression.

However, this paradigm has been predominantly built from the targeted analysis of specific cell populations and cytokines in KO mice or through flow-cytometry analyses, and the relative contribution of the multiple mechanisms to parasite control involved is unclear. Also, the contribution of innate immunity during the chronic phase of *T*. *cruzi* infection is often ignored, although a few studies suggest a role of innate mechanisms in the progression of chronic Chagasic cardiomyopathy (12–17). Thus, nontargeted approaches such as transcriptomic studies of patients with different clinical profiles have provided more integrated information and suggested that NK/CD8^+^ T cell cytotoxicity could play a key role in determining disease progression (18).

In addition, *T*. *cruzi* genetic diversity has also been thought for a long time to contribute to differences in disease progression and severity (19, 20), as major biological differences among strains can be observed both in vitro and in vivo (21, 22). However, the generalization of these findings has been challenging and the association between parasite discrete typing units (DTUs), the main genetic lineages structuring *T*. *cruzi* diversity, with disease progression has remained elusive (23, 24, 25).

Monitoring macaques with natural *T*. *cruzi* infection has shed new light on some of these aspects, as these animals recapitulate human responses to infection well (26–28). Indeed, a clear association could be detected between chronic disease progression, defined by blood parasite levels and changes in electrocardiographic (ECG) recordings, and the diversity of infecting parasite strains (29), as a large proportion of natural infections include multiple strains. Thus, infections with a low diversity of strains were associated with increased blood parasite burden over time and changes in ECG profiles indicative of early cardiac disease progression, while infections with a high diversity of strains were associated with a better control of blood parasites and the absence of significant changes in ECG profiles (29). These observations suggest that differences in host-parasite relationships according to parasite diversity may contribute to disease progression. As a first step to better characterize host responses, we evaluated here the transcriptomic response of unstimulated Peripheral blood mononuclear cells (PBMCs) from naturally infected macaques and tested for potential heterogeneity in their gene signatures according to disease progression and parasite diversity. Such an integrative approach can provide a comprehensive view of some of the mechanisms underlying Chagas disease pathogenesis.

## Results

### Differential PBMC gene signature from T. cruzi–infected and uninfected macaques.

**Figure 1:**
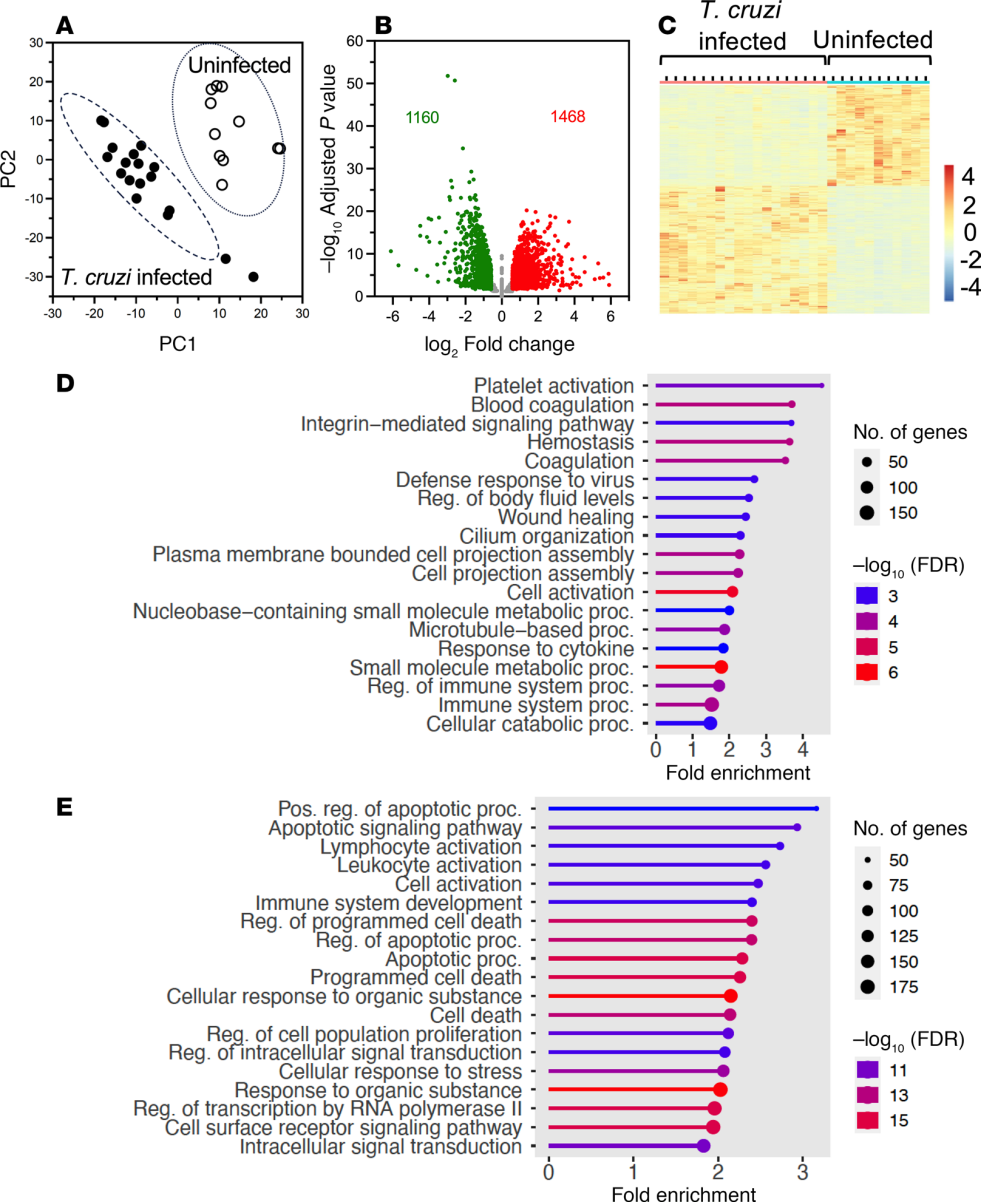
Differences in gene expression profile between *T*. *cruzi*–infected and uninfected macaques. (**A**) PCA plot of PBMC gene expression profiles. Normalized adjusted read counts were used as the inputs for PCA to visualize differences in gene expression profiles among samples. (**B**) Volcano plot of differential gene expression between uninfected and infected macaques. In total, 2,628 genes were differentially expressed (adjusted *P* < 0.05, fold change > 1.5) in PBMCs from *T*. *cruzi*–infected and uninfected macaques, with 1,468 upregulated (red) and 1,160 downregulated genes (green). (**C**) Heatmap of differentially expressed genes, which differences in expression level is color-coded as indicated in the color scale (log_10_ fold change). Note the clustering of up- and downregulated genes in PBMCs from infected and uninfected macaques. (**D**) Lollipop graph of significantly upregulated pathways in infected macaques. (**E**) Lollipop graph of significantly downregulated pathways in infected macaques. Significance based on PAGE with FDR.

We used PBMCs from 29 macaques, including 11 uninfected controls and 18 macaques with natural *T*. *cruzi* infection (Table 1) for a cross-sectional study of their unstimulated transcriptomic profile. These had been infected with *T*. *cruzi* for 3–7 years, as determined by the approximate time of seroconversion, and were on average 11 years old (range, 4–19). We performed RNA-Seq to obtain about 20 million reads per sample that were mapped to the Mmul10 macaque reference genome. Batch-adjusted, filtered read counts were used to call differentially expressed genes between groups. A clear clustering of the 2 macaque groups was observed through principal component analysis (PCA), indicating that chronic *T*. *cruzi* infection could explain part of the variation in gene expression among samples (Figure 1). Based on a mean expression fold change > 1.5 between groups, there were 1,468 genes significantly upregulated and 1,160 genes significantly downregulated between uninfected and infected macaques (Figure 1B). Clustering of individual macaques according to *T*. *cruzi* infection status was further observed based on differentially expressed genes (Figure 1C).

To explore the function of the differentially expressed genes, upregulated and downregulated genes were queried for Gene Ontology (GO) and Kyoto Encyclopedia of Genes and Genomes (KEGG) pathways using overrepresentation analysis. Among infected macaques, upregulated pathways included platelet activation, blood coagulation, integrin-mediated signaling pathway, defense response to virus, cell activation, and immune system processes (Figure 1D). Downregulated pathways included multiple apoptotic signaling pathways/apoptotic processes, lymphocyte/leukocyte activation, immune system development, and both intracellular and cell surface signaling pathways (Figure 1E). These are in general agreement with extensive alterations of immune cell activation and differentiation in response to the ongoing chronic infection. Gene set enrichment analysis (GSEA) highlighted some similar pathways differentiating uninfected and infected macaques (Supplemental Figure 1; supplemental material available online with this article; https://doi.org/10.1172/jci.insight.186003DS1), but only platelet activation and TNF-α pathways had some statistical support, possibly due to extensive heterogeneity in individual macaque responses to infection.

### Immune profile of T. cruzi–infected macaques.

**Figure 2:**
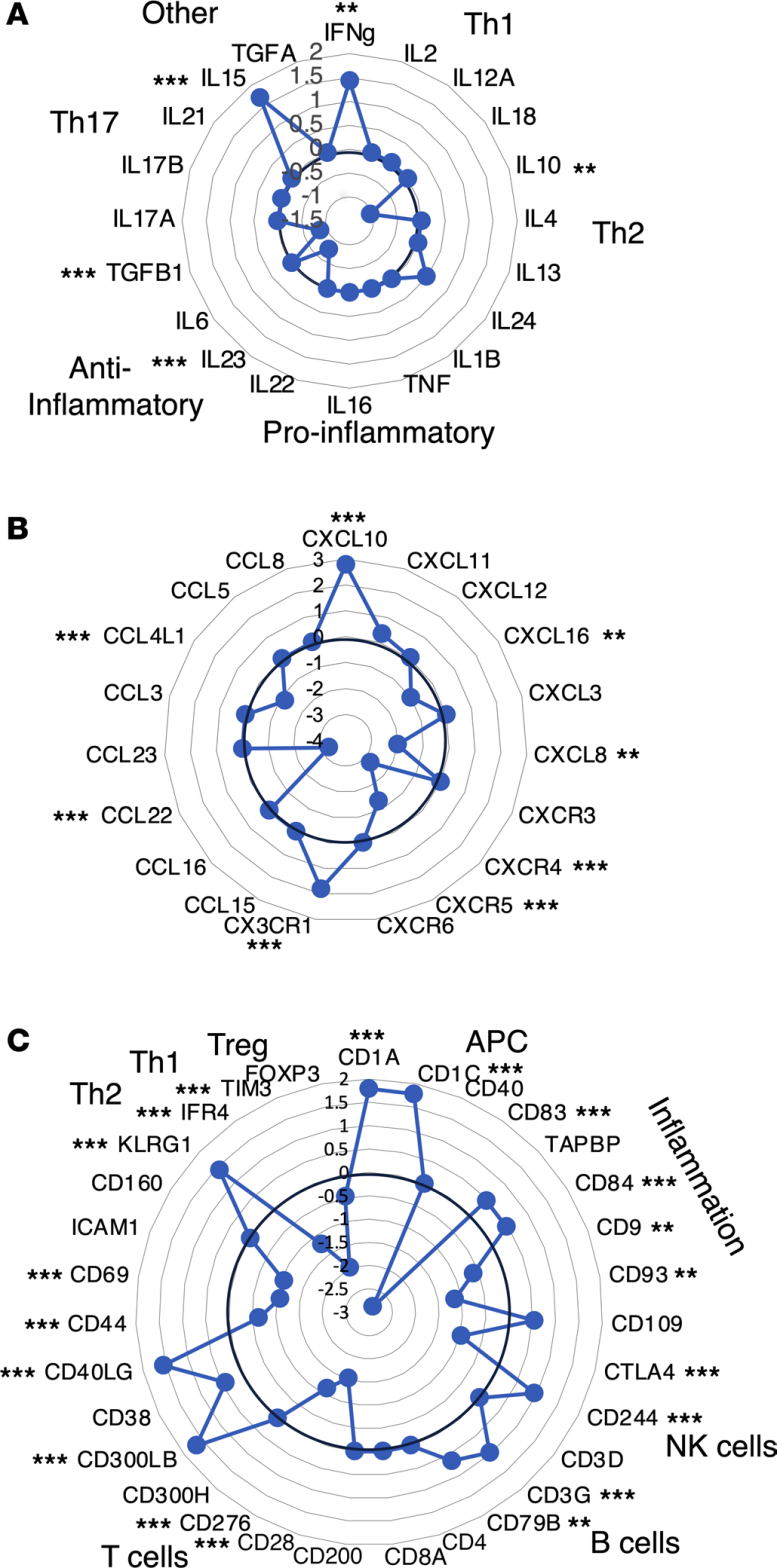
Cytokines, chemokines, and immune cell marker expression levels. Radar plots of the expression level for the indicated genes, expressed as the log_2_ fold difference between PBMCs from *T*. *cruzi*–infected and uninfected macaques, are shown. The thicker line at 0 indicate no difference in expression level. (**A**) Cytokines. (**B**) Chemokines and chemokine receptors. (**C**) Immune cell markers. Adjusted *P < 0.05, **P < 0.01, ***P < 0.001 by Wald test as implemented in DESeq2.

To better characterize the immune profile of *T*. *cruzi*–infected macaques, we focused on the expression profile of cytokine, chemokines, and immune marker genes, in an integrated manner. *IL15* and *IFNG* genes were upregulated in infected macaques, while TGF-β1 (*TGFB1*), *IL23*, and *IL10* genes were downregulated, and other cytokines appeared unaffected (Figure 2A). Infection was also associated with the marked upregulation of CXC motif chemokine ligand 10 (*CXCL10*) and CX3C motif chemokine receptor 1 (*CX3CR1*) genes, associated with CD8^+^ T cell response and the recruitment of NK cells, respectively, while C-C motif chemokine ligand 22 (*CCL22*), CXC motif chemokine ligand 8 (*CXCL8*), CXC motif chemokine receptor 4 (*CXCR4*), and *CXCR5* genes, which are involved in the chemotaxis and migration of T cells and neutrophils, were downregulated (Figure 2B). Analysis of additional markers further indicated a mixture of activation and inhibition of several immune cell populations (Figure 2C). These included antigen presenting cells, with the upregulation of *CD1C* and TAP binding protein (*TAPBP*) genes in infected macaques, while *CD83* was downregulated and *CD40* remained expressed at the same level. There was also a mixture of upregulated antiinflammatory marker genes (*CD84*) together with downregulated markers (*CD9*, *CD93*, cytotoxic T-lymphocyte associated protein 4 [*CTLA4*]). B cell marker *CD79B* was upregulated, while no difference was detected in *CD4* or *CD8* expression. Lymphocyte activation marker CD40 ligand (*CD40LG*) gene was upregulated, while *CD69* was downregulated. T cell exhaustion/senescence marker *CD160* was not differentially expressed, while killer cell lectin-like receptor G1 (*KLRG1*) was upregulated in PBMCs from infected macaques and costimulatory receptor *CD28* gene was downregulated. IFN regulatory factor 4 (*IFRA4*) and T cell immunoglobulin and mucin-domain containing 3 (*TIM3*), markers for Th2 and Th1 cells, respectively, were downregulated, and Treg marker forkhead box P3 (*FOXP3*) tended to be downregulated as well.

**Figure 3:**
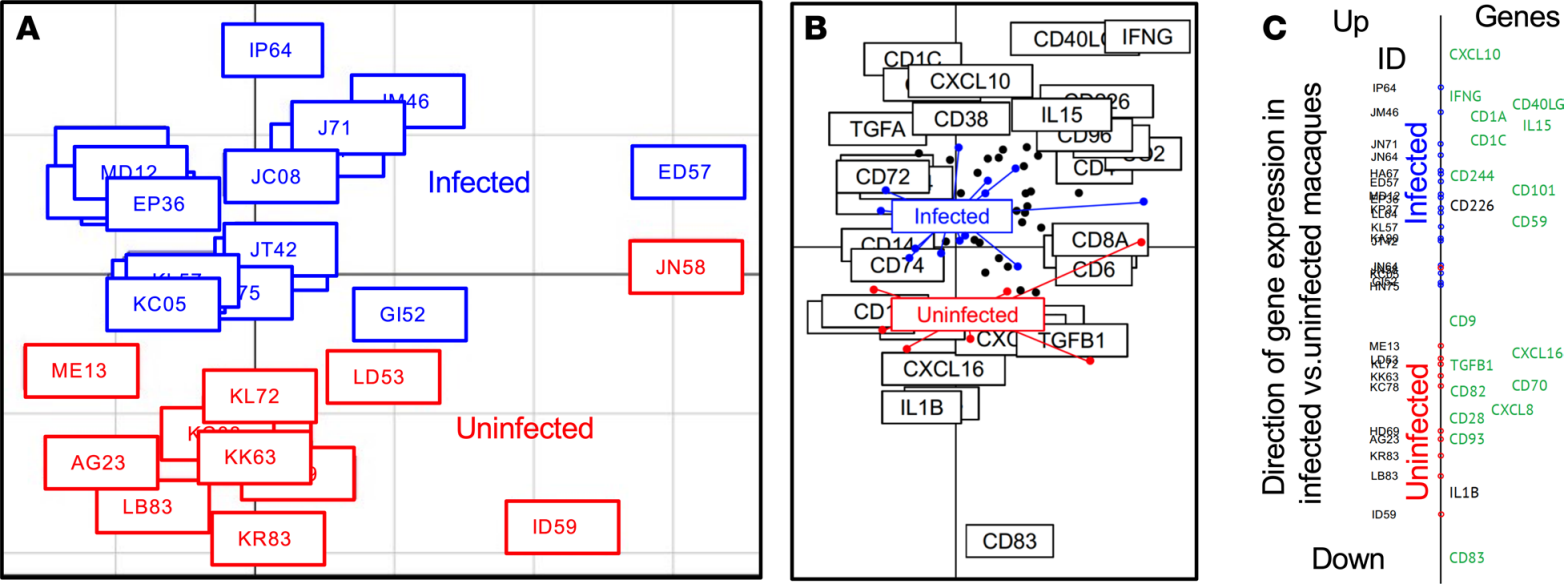
Multivariate analysis of PBMC gene expression between *T*. *cruzi*–infected and uninfected macaques. (**A**) BGA of infected and uninfected macaques, indicating clustering of individual macaques. Infected macaques are indicated in blue, and uninfected are indicated in red. (**B**) Overlay of macaque clustering and gene clustering from BGA. Colored dots and lines represent individual macaques; infected are indicated in blue, and uninfected are indicated in red. Black dots and boxed gene names represent individual genes, with genes found at the ends of the main BGA discrimination axis representing genes most associated with macaque groups. (**C**) Top 20 genes which expression profile is most associated with each macaque group. Genes in the top part of the graph are upregulated, while those at the bottom are downregulated in infected macaques versus uninfected macaques. A significant difference in gene expression level was detected by DESeq2 analysis for the genes labeled in green. Statistical test used was Wald test as implemented in DESeq2.

Next, multivariate analysis was used to identify immune markers most associated with chronic infection. Between-group analysis (BGA) of gene expression revealed strong separation of chronically infected and uninfected macaques along the main BGA discrimination axis (Figure 3A). By overlaying coordinate data of the samples over the clustering of gene expression, genes found at the ends of the main BGA discrimination axis represents genes most associated with groups (Figure 3B). The top 20 genes associated with chronic *T*. *cruzi* infection in macaques were identified along the main BGA discrimination axis (Figure 3C), most of which had also been identified as differentially expressed by DESeq2 analysis. Infection was associated with the upregulation of proinflammatory genes *CXCL10*, *IFNG*, and *IL15*, alongside several NK and T cell activation markers such as *CD244*, *CD40LG*, and *CD226*. The *CD1C* marker of DCs was upregulated, as was *CD59*, which mediates complement-mediated cell lysis and lymphocyte signaling. On the other hand, there was a downregulation of the proinflammatory cytokine genes *IL1B* and of the antiinflammatory *TGFB1*, alongside chemotactic molecules genes *CXCL8* and *CXCL16*. Both inflammation marker *CD93* and antiinflammation marker *CD9* were downregulated. There was also a downregulation of activation markers *CD70*, *CD82*, and *CD83*, suggesting altered Treg function and/or incomplete activation of T and B cells, and costimulatory molecule *CD28* gene was also downregulated. Together, these data suggest that chronically infected macaques present a mixture of immune activation to target *T*. *cruzi* parasites, together with immune suppression to prevent uncontrolled tissue inflammation and damage, with the involvement of both innate and adaptive immune mechanisms and important heterogeneity in individual responses.

### Differential PMBC gene signature from controller and progressor macaques.

**Figure 4:**
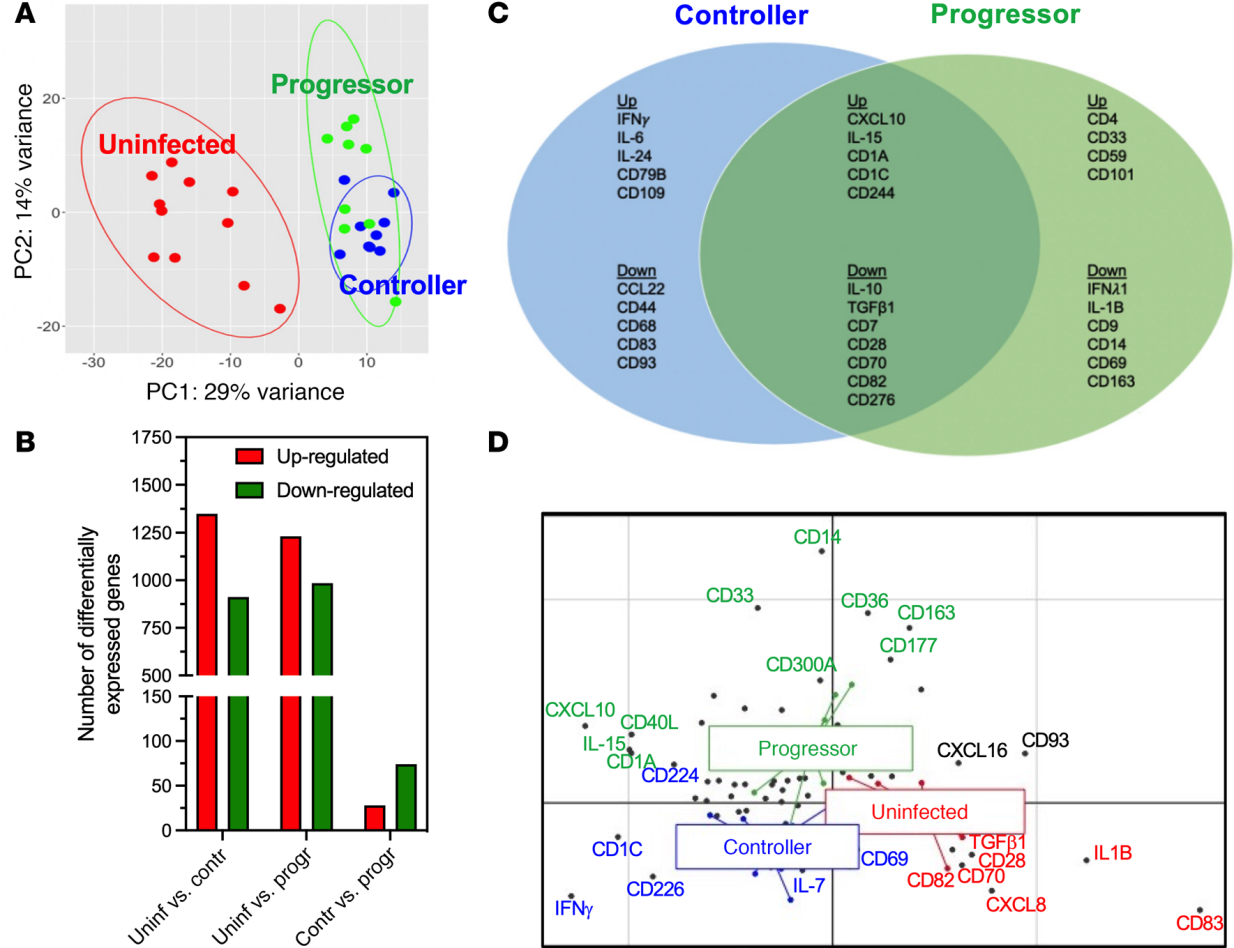
PBMCs gene signature associated with *T*. *cruzi* parasite control. (**A**) PCA of gene expression profile of PBMCs from uninfected, progressor, and controller macaques. (**B**) Number of statistically significant (adjusted *P* < 0.05) differentially expressed genes of PBMCs from uninfected, progressor, and controller macaques. (**C**) Venn diagram of statistically significant (adjusted *P* < 0.05) differentially expressed genes associated with the immune response of PBMCs from controller and progressor macaques. Shared and unique up- and downregulated genes from the 2 groups are indicated. (**D**) BGA multivariate analysis of the gene expression signatures differentiating the 3 groups of macaques. Colored dots and lines represent individual macaques, colored as controller (blue), progressor (green) and uninfected (red). Black dots represent individual genes, with gene labels colored based on which macaque group they are associated with. Statistical test used was Wald test as implemented in DESeq2.

Because there is extensive variability in disease progression and severity in *T*. *cruzi*–infected hosts, we next tested for differences in gene expression profiles among macaques with progressive or controlled infection, based on changes in their parasite burden (Table 1). PCA of gene expression profile revealed a differential clustering of progressor, controller, and uninfected macaques (Figure 4A), with both infected groups presenting a large number of differentially expressed genes with uninfected macaques, as well as 28 significantly upregulated and 74 downregulated genes between progressor and controller macaques (Figure 4B). These data were in agreement with a different host response in progressor and controller macaques. Focusing on genes involved in the immune response, we detected a set of genes significantly up- and downregulated in both groups of infected macaques, including the upregulation of *IL15*, *CXCL10*, and *CD244* and the downregulation of *IL10*, *TGFB1*, or *CD28* genes (Figure 4C). On the other hand, some genes were specifically modulated in PBMCs from controller and progressor macaques, respectively. For example, *IFNG*, *IL6*, and *IL24* genes were upregulated in controller macaques, together with B cell marker *CD79B* gene as well as antiinflammatory marker *CD109*, while the *CCL22* gene involved in the chemotaxis and migration of T cells was downregulated, together with activation marker *CD83* (Figure 4C), suggesting an active adaptative response with a controlled inflammation. Conversely, PBMCs from progressor macaques presented an upregulation of *CD4*, as well as of *CD59*, which inhibits complement-mediated cell lysis to protect cells from complement damage, and *CD101*, which has been proposed as a marker of pathological responses to dysbiosis; they also presented a downregulation of *IFNL1*, which plays a role in viral and bacterial infections, as well as the proinflammatory cytokine gene *IL1B*. Macrophage antiinflammatory markers *CD9* and *CD163*, and lymphocyte activation marker *CD69*, were also downregulated in progressor macaques (Figure 4C), suggesting a more proinflammatory response. For an integrated analysis of the immune response, we again performed a multivariate analysis of the gene expression profile among macaque groups (Figure 4D), which confirmed the unique gene signatures of PBMCs from controller and progressor macaques.

### Differential PBMC gene signatures reflect T. cruzi strain diversity.

**Figure 5:**
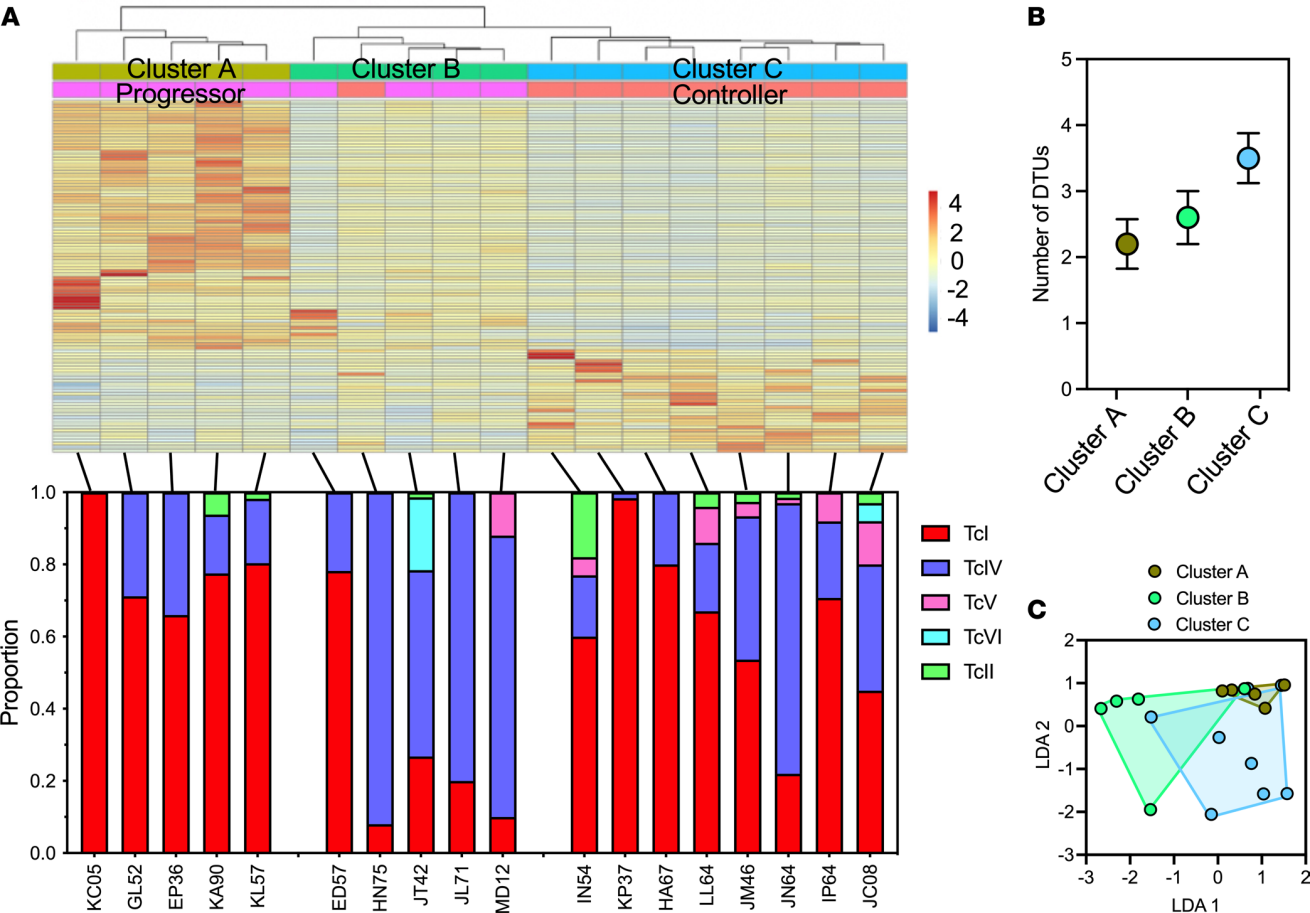
Unsupervised clustering of infected macaques based on PBMC gene signatures. (**A**) Heatmap represents expression level of differentially expressed genes, color coded as indicated (log_2_ fold change). Hierarchical clustering of infected macaques resulted in 3 clusters, labeled as A, B, and C, that show some overlap with progressor/controller classification: cluster C comprise only controller macaques, while clusters A and B include progressor macaques and 1 controller macaque that was assigned to cluster B. Bottom panel shows *T*. *cruzi* parasite DTU composition among individual macaques from the respective gene expression clusters. (**B**) Average number of DTUs infecting individual macaques from the respective clusters. Data presented as mean ± SEM. (**C**) LDA of macaques based on the DTU composition of infecting parasite strains, indicating significant clustering corresponding to that based on PBMC gene signatures (1-way PERMANOVA, *P* = 0.012), with up to 77% of individual macaques correctly classified to their respective cluster.

To further understand the heterogeneity of host responses to *T*. *cruzi* infection, we next performed an unsupervised clustering of infected macaques, based on their PBMC differential gene expression profiles. This analysis indicated the presence of 3 clusters of unique gene signatures among infected macaques (Figure 5A). Clusters A and B included mostly macaques that had been classified as progressors, although they presented clear differences in their gene expression profiles, while cluster C comprised controller macaques. One macaque (HN75), initially classified as controller, rather clustered with progressor macaques from cluster B. Remarkably, this clustering of macaques based on their transcriptomic response to *T*. *cruzi* infection matched their parasite strain profiles well. Indeed, macaques from cluster A were predominantly infected by TcI parasites, while those from cluster B were predominantly infected with TcIV parasites, and those from cluster C were infected with the largest diversity of strains that included mixtures of TcI, TcII, TcIV, TcV, or TcVI in variable proportions (Figure 5, A and B). Linear discriminant analysis (LDA) of parasite strain composition was also able to significantly discriminate the 3 clusters (1-way permutation ANOVA [PERMANOVA], *P* = 0.01), and over 77% of macaques could be correctly reclassified into their corresponding gene expression profile group based solely on *T*. *cruzi* parasite composition data (Figure 5C). These results strongly suggest that infecting *T*. *cruzi* strain composition was a major determinant of host response and disease progression.

**Figure 6:**
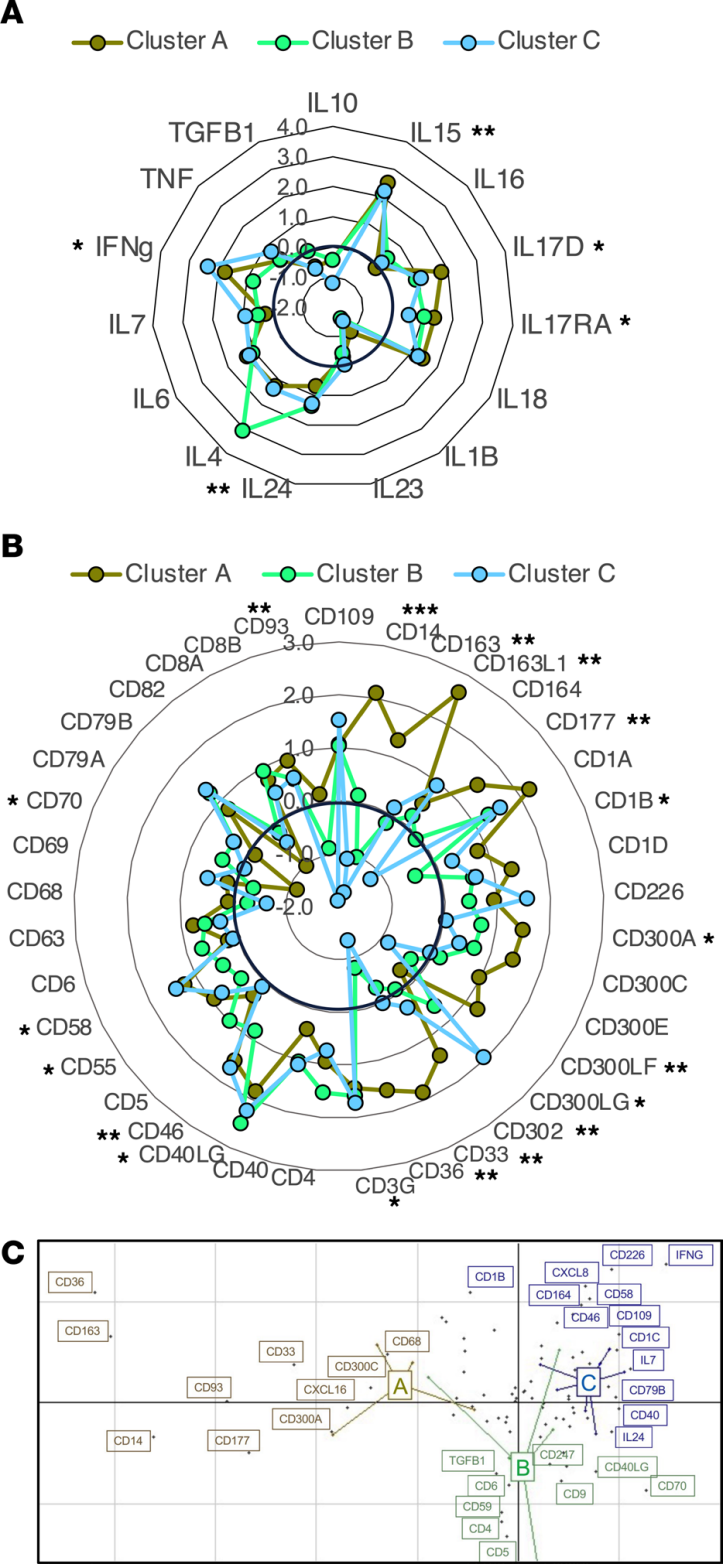
Immune profile associated with *T*. *cruzi*–infected macaques. (**A** and **B**) Cytokine (**A**) and immune marker (**B**) differential expression levels among clusters A, B, and C of infected macaques. Radar plots indicate differential expression of the indicated genes, as log_2_ fold change. Thicker black circle corresponds to no change. *P < 0.05, **P < 0.01, and ***P < 0.001. (**C**) BGA multivariate analysis of PBMC gene signatures among the 3 clusters of macaques. Colored dots and lines represent individual macaques, colored as cluster A (brown), cluster B (green), and cluster C (blue). Black dots represent individual genes, with gene labels colored based on which macaque group they are associated with. Statistical test used was Wald test as implemented in DESeq2.

We further assessed differences in immune profile among the 3 clusters of infected macaques. Analysis of cytokine and immune marker gene expression indicated clear differences in the immune response of each infected macaque group (Figure 6, A and B). Thus, the PBMC response of progressor macaques from cluster A was characterized by an increased expression of *IFNG*, *IL15*, and *IL17* cytokine genes, together with the upregulation of *CD163* associated with alternatively activated macrophages and *CD1A* associated with DCs. At the same time, the upregulation of *CD300A* and *CD300E* and the downregulation of *CD82* genes suggested the inhibition of effector functions and T cell responses. Furthermore, *CD177* and *CD33* were upregulated, in agreement with poor pathogen control as observed in SARS-Cov2 and HIV infections (30, 31).

PBMCs of progressor macaques from cluster B presented downregulated expression of *IFNG*, and even though the upregulation of *CD40LG* suggested T cell activation, this may be dampened by the upregulation of *CD5* and *CD6* genes, which mediate the control of aberrant immune responses and inhibit T cells, respectively (Figure 6, A and B). Furthermore, the downregulation of *CD93* suggested a low immune infiltration.

Lastly, PBMCs from controller macaques from cluster C also presented a high level of *IFNG* expression, together with the upregulation of *IL15* and *IL17* cytokine genes, but the upregulation of *CD300LG* suggested higher lymphocyte migration, while the downregulation of *CD177* was in agreement with improved pathogen control (Figure 6, A and B).

These differences in immune response profiles among the 3 groups of *T*. *cruzi*–infected macaques were also largely supported by a BGA of the gene expression profiles (Figure 6C). Thus, progressor macaques from cluster A were characterized by *CD163*, *CD177*, *CD36*, *CD33*, and *CXCL16* gene expression, which associate with a proinflammatory response; those from cluster B were associated with the expression of *CD5*, *CD59*, *CD247*, and *TGFB1* genes, suggesting more regulatory processes. On the other hand, the gene profile from controller macaques from cluster C included genes such as *IFNG*, *CD226*, *CD79B*, *CD1C*, and *CXCL8*, suggesting a broad activation of B and T cells as well as from innate cells, and the expression of *CD109* and *CD40* genes also suggested some regulatory processes (Figure 6C).

**Figure 7:**
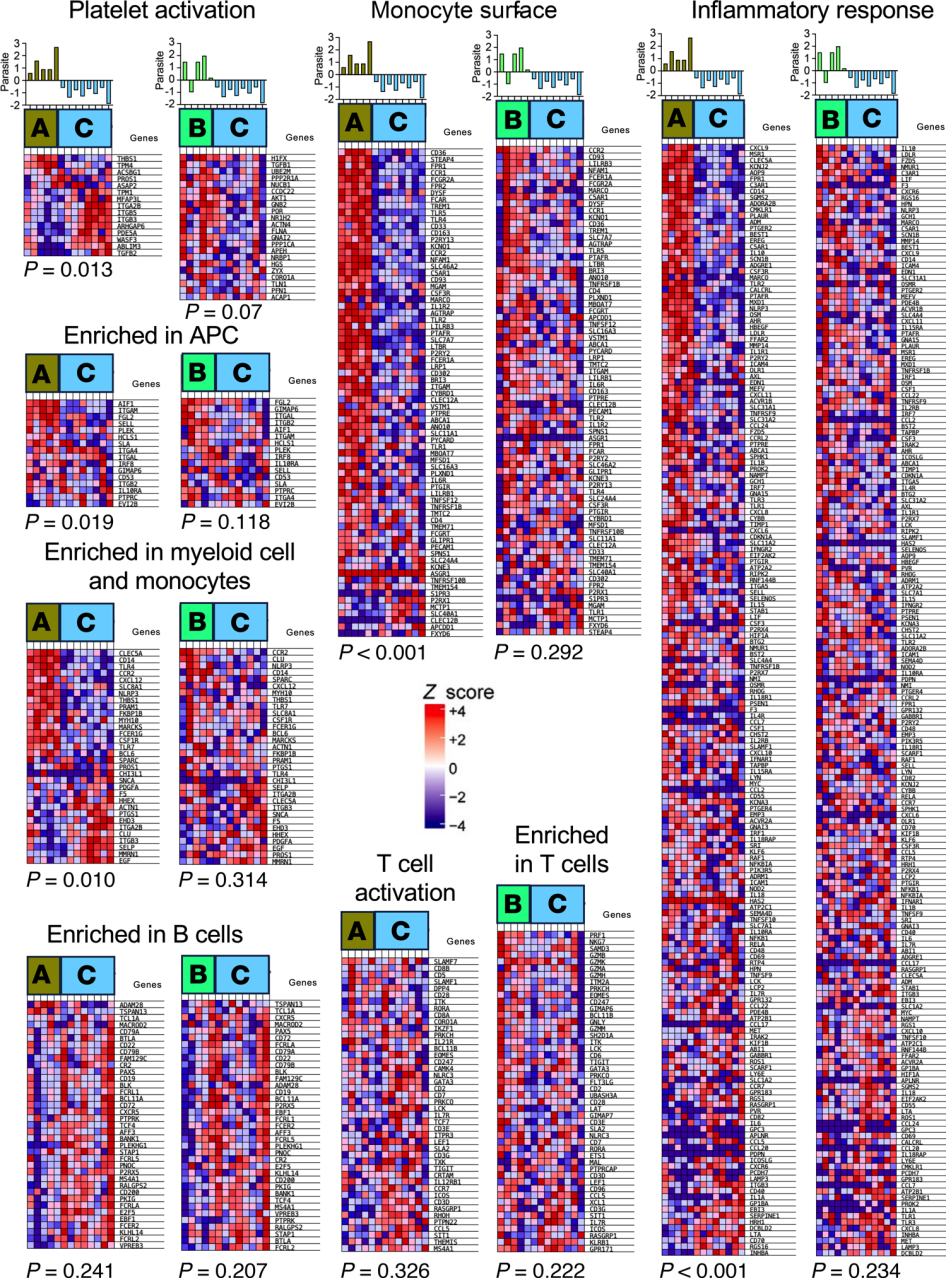
GSEA pathways associated with macaque PBMC responses. The indicated pathways from the BTM and Hallmark modules were analyzed among progressor macaques from clusters A and B, compared with controller macaques from cluster C. Each column represent an individual macaque. The top bar graphs represent changes in blood parasite burden overtime, expressed in log_2_ values, with positive values indicating an increase and negative values a decrease in parasite burden. The statistical significance of differences in gene expression profile between the indicated macaque clusters is indicated at the bottom of each heatmap. Statistical test is as implemented in GSEA.

GSEA further identified multiple pathways that were differentially associated with each cluster of macaques. Again, gene signatures from progressor macaques from cluster A presented the most proinflammatory responses, predominantly involving innate immune processes mediated by antigen presenting cells and monocytes and a downregulated platelet activation pathway, while pathways associated with B and T cell adaptive responses were marginally affected compared with controller macaques from cluster C (Figure 7). This response also included exacerbated TLR and inflammation pathways, as well as upregulated blood coagulation pathway compared with cluster C macaques (Supplemental Figure 2).

Progressor macaques from cluster B also tended to present a proinflammatory innate component, although not as marked as cluster A, and an upregulated platelet activation pathway (Figure 7 and Supplemental Figure 3), with some upregulation of B cell markers compared with cluster A (Supplemental Figure 3). On the other hand, gene expression from pathways associated with B and T cells was not significantly different between cluster B and C macaques, except for some upregulation of mitotic CD4 cells in cluster C macaques (Supplemental Figure 4). Thus, controller macaques from cluster C presented the most antiinflammatory response and a controlled innate response compared with clusters A and B.

**Figure 8:**
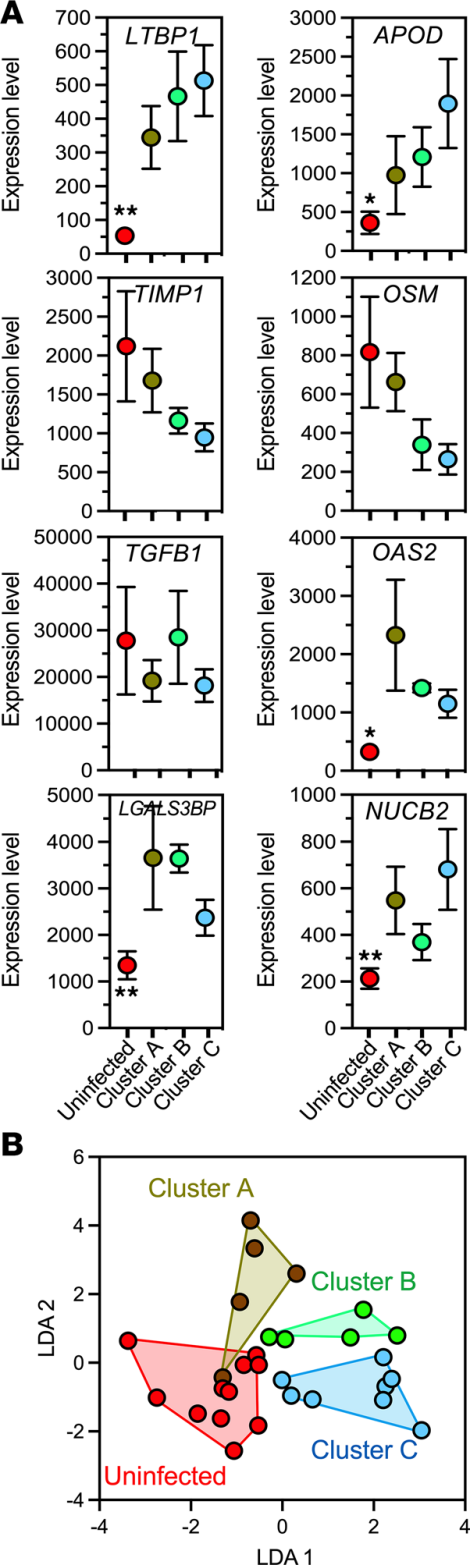
Biomarkers for differential host response to *T*. *cruzi* infection. (**A**) Expression level of the indicated genes as potential biomarkers of the host response/parasite strain composition expressed as mean normalized counts ± SEM. One-way ANOVA, *P < 0.05 and **P < 0.01. (**B**) LDA of candidate biomarkers indicating a significant discrimination among macaque groups based on the signature of 8 biomarkers (1-way PERMANOVA, *P* = 0.007), with 25 of 29 (86.2%) of individuals correctly classified in their respective group.

Finally, we assessed the potential of some of the expressed genes to serve as biomarkers of host immune response profile and disease progression. Several potential biomarkers were specifically up- or downregulated in PBMCs of the different macaque groups (Figure 8A). The combination of only 8 genes, including latent transforming growth factor β binding protein 1 *(LTBP1*), apolipoprotein D (*APOD*), TIMP metallopeptidase inhibitor 1 (*TIMP1*), oncostatin M (*OSM*), TGF-β 1 (*TGFB1*), 2′-5′-oligoadenylate synthetase 2 (*OAS2*), galectin 3 binding protein (*LGALS3BP*), and nucleobindin 2 (*NUCB2*) genes, was sufficient to correctly discriminate among the 3 clusters of infected macaques, as indicated by LDA analysis (1-way PERMANOVA, *P* = 0.002), and 25 of 29 (86.2%) macaques could be correctly reclassified based on the signature of these markers (Figure 8B).

## Discussion

Host responses to *T*. *cruzi* infection can lead to a broad range of clinical manifestations, ranging from an asymptomatic status to severe cardiac and digestive disease (3, 32). Many studies have focused on specific components of the immune response to identify cell populations, cytokines, and immune processes involved, but integration of this knowledge has been challenging, and the relative contribution of the multiple immune components for parasite control and disease progression remains unclear (33, 34). Here we used RNA-Seq of PBMCs from naturally infected macaques to investigate the transcriptomic response of these cells in an integrated perspective.

As expected, we found that chronic *T*. *cruzi* infection was associated with major alterations of PBMC gene expression profiles, characterized by a mixture of proinflammatory immune activation, together with immune suppression, which may reflect control of hyperpolarized inflammation and/or immune exhaustion associated with parasite persistence and insufficient control. This is in general agreement with a broad adaptive immune response observed in the chronic phase of infection (9, 11). Our integrated approach also points to the important contribution of innate immune processes such as platelet activation, as well as hemostasis and blood coagulation. Indeed, hypercoagulability and thrombosis have been involved in the chronic phase, independently of the development of Chagasic cardiomyopathy (13, 35–37). Altered platelet activation has also been described in mice experimentally infected with *T*. *cruzi* (38), as well as in patients (14). In addition to their role in hemostasis and thrombosis, platelets can also play an immunomodulatory role in infection through multiple mechanisms (39, 40). While the role of innate responses in the early steps of *T*. *cruzi* infection have been described, the continued involvement of innate processes after years of infection is more striking (5, 17, 41).

Remarkably, our results also shed light on some of the key mechanisms underlying the diversity of host responses to *T*. *cruzi* infection, which has been a major hurdle in understanding Chagas disease progression. Indeed, major differences in the transcriptomic response of host PMBCs were detected according to whether or not these hosts were able to control parasite multiplication. Furthermore, while controller macaques were a fairly homogenous group based on their gene expression profile, progressor macaques presented additional heterogeneity in their profiles, with 2 main gene signatures, suggesting that there are multiple ways to have an inadequate immune response against *T*. *cruzi* (clusters A and B of progressor macaques) but likely less options to correctly control the parasite (cluster C of controller macaques). Strikingly, these differences in host responses could be explained in large part by the composition of infecting parasite strains, with progressor macaques from cluster A infected predominantly with TcI and those from cluster B with TcIV, while controller macaques from cluster C were infected with a high diversity of strains, including TcI, TcII, TcIV, TcV, or TcVI, in variable proportions. This expands previous work hinting at differences in cytokine profiles in patients infected with TcI, TcII, or mixtures of TcI and TcII (34), and it strongly supports the hypothesis that disease progression may be predicted based on parasite diversity as proposed before (29). Importantly, this implies that such infections with multiple parasite strains occur at once rather than sequentially, which seems supported by observations in triatomine vectors (42, 43) and the low likelihood of sequential infections (44).

Analysis of the gene expression profile heterogeneity among *T*. *cruzi*–infected macaques further revealed key features of the immune response during the chronic phase in an integrated manner. Thus, macaques predominantly infected with TcI had the most proinflammatory responses compared with controller macaques infected with a high parasite diversity. Strikingly, many of the enriched pathways in these progressor macaques were associated with the innate immune response, with alterations in platelet, monocyte, myeloid cells and NK cell activation, and TLR pathways, rather than with the adaptive immune response, as there was limited enrichment in B or T cell–associated pathways. On the other hand, controller macaques displayed a strong adaptative response but in a less proinflammatory environment. These results are in agreement with a comparable study in patients with Chagas disease, as these displayed blood gene signatures from several pathways associated with innate immunity such as TLR signaling and monocyte and NK cell responses that were associated with different levels of severity of chronic cardiomyopathy, although no information was available on *T*. *cruzi* strains infecting these patients (18). Thus, while adaptive immunity is clearly needed (9, 11) and can play a role in Chagas disease progression as observed before (45), our results suggest that innate immunity may be a predominant contributor to pathogenesis, even though it is often overlooked in chronic infections. More specifically, it is likely that trained immunity can be responsible for differences in the inflammatory environment. Indeed, trained immunity involves the long-term reprogramming of immune cells including monocytes, macrophages, and/or NK cells, through epigenetic modifications (46). This training/programming of the innate cells may occur during the acute phase of infection and vary according to *T*. *cruzi* strain diversity, to condition the subsequent inflammatory environment and the adaptive response during the chronic phase (47) and, thus, mediate pathogenesis. Trained immunity has been implicated in host response to pathogens such as *Mycobacterium* (48) and can also mediate nonspecific effects of vaccines against nontarget pathogens, such as the effect of BCG vaccination in modulating clinical and immune responses to *Plasmodium falciparum* infection (49), through the reprogramming of neutrophils (50). Remarkably, memory-like NK cells generated in response to vaccination of mice with a highly attenuated *T*. *cruzi* strain have recently been found to be critical for protection against a secondary infectious challenge, strengthening the role of trained innate immunity in long-term parasite control (51).

Finally, we also identified a simple biomarker signature that can discriminate among host transcriptomic response profiles and that also reflects *T*. *cruzi* strain diversity and disease progression. It may have important clinical relevance to identify patients most at risk of progressing to chronic cardiomyopathy (52). Previous studies identified several clinical parameters as predictors of *T*. *cruzi* PCR positivity in patients (53), and clinical parameters from cardiac echography together with brain natriuretic peptide (BNP) levels but not those of TGF-β1 have been associated with Chagas disease cardiovascular events (54). However, TGF-β serum levels increased in experimentally infected mice and correlated well with cardiac fibrosis and echographic alterations (55). Further validation of these biomarkers may lead to new tools for prognosis of Chagas disease.

A limitation of our study is that transcriptomic responses were only assessed at a single time point, and no longitudinal assessment of individual responses was performed. Such studies would be key to document transcriptomic changes as disease progresses but may be difficult to perform, given the slow progression of clinical disease. The limited extent of cardiac disease in our cohort of macaques is also another limitation, as none of the animals presented severe cardiomyopathy, which may be associated with additional transcriptomic changes. Indeed, all appeared to have normal ECGs, with only statistical differences detectable through multivariate analysis of ECG parameters (29), suggesting very early stages of cardiac alterations. Nonetheless, stratification of progressor/controller macaques based on changes in blood parasite burden remained helpful to assess the heterogeneity of individual responses to infection. Nonetheless, we still detected some inconsistencies among individual macaque transcriptomic responses, likely due to additional factors that may further modulate host response and the progression of the infection, including host genetics, host status at the time of infection, and infecting parasite dose, but these could not be taken into account.

In conclusion, our integrative study demonstrates that *T*. *cruzi* parasite diversity is a major determinant of host response to natural infection observed during the chronic phase in macaques, leading to differences in parasite control and disease progression. While differences in adaptive immune responses can be observed according to *T*. *cruzi* diversity, more striking alterations of innate immune processes can be detected, suggesting that trained immunity is a major component of Chagas disease pathogenesis. A likely hypothesis is that the initial innate response to *T*. *cruzi* infection is conditioned by parasite strain diversity, resulting in different profiles of trained immunity that modulate the adaptive immune response, ultimately shaping parasite control or its persistence during the chronic phase. These results call for further characterization of the cross-talk between innate and adaptive immunity and how altered trained immunity contributes to Chagas disease pathogenesis, as this may lead to better treatments and more effective vaccines.

## Methods

### Sex as a biological variable.

Infected macaques of both sexes were included in the study as indicated in Table 1, and similar findings are reported for both sexes.

### Naturally infected macaques.

Chagasic macaques were part of a cohort of naturally infected rhesus macaques (*Macaca mulatta*) held at the Tulane National Primate Research Center with confirmed *T*. *cruzi* infection (29). Eighteen animals were included in this study. These had been infected with *T*. *cruzi* for 3–7 years, as determined by the approximate time of seroconversion, and were on average 11 years old (range, 4–19) (Table 1). An additional 11 uninfected healthy control macaques were included for comparison. Whole blood was collected with EDTA-K2, and ECG recording was performed during routine veterinary care of the animals and represent an opportunistic sample. A cross-sectional study of unstimulated PBMC transcriptome was performed at a single time point.

Disease progression was assessed by measuring blood parasite burden by quantitative PCR (qPCR) as described before (29), every 6–8 months over a 24- to 48-month period. Changes in blood parasite burden over time were calculated, and macaques were classified as controller when presenting a decreasing blood parasite burden over time or as progressor when presenting an increase in blood parasite burden (Table 1). *T*. *cruzi* parasites infecting the macaques were genotyped by next-generation sequencing of the miniexon marker as before (29), to identify parasite DTUs present and their relative proportion. Cardiac function was also monitored by ECG recordings and analysis of the main wave patterns (29). Specific abnormalities/arrythmias were not observed, and all ECGs were normal; no macaque presented with severe cardiac disease. However, most macaques in the progressor group presented significant changes in ECG profile over time, suggesting early conduction defects, while most in the controller group had no significant change over time (Table 1). BNP plasma concentration was measured by ELISA (RayBiotech Inc.) and tended to be elevated in progressor macaques, although this did not reach statistical significance (Kuskal-Wallis, *P* = 0.22; Supplemental Figure 5).

### RNA extraction from PBMCs and RNA-Seq.

PBMCs were isolated from whole blood centrifugation at 400*g* with a Ficoll-Paque (MilliporeSigma) solution, washed with RPMI and PBS, and cryopreserved in Cryostor (Biolife Solutions) solution until use. Frozen PBMCs were thawed and assessed for viability through counting in a hemocytometer with trypan blue dye staining. Thawed unstimulated PBMCs with at least 90% viability were used for RNA extraction. RNA was extracted using the PerfectPure RNA Cultured Cell Kit (5 Prime) as per manufacturer instructions, and RNA integrity was assessed with an Agilent Bioanalyzer; all samples had a RNA integrity number (RIN) > 8. About 100–200 ng of RNA per sample was used for library preparation and sequencing on an Illumina MiSeq platform.

### Differentially expressed gene calling.

Data analysis and visualization was performed using R version 4.2.1 (56). Reads were filtered for quality and mapped to the rhesus macaque Mmul10 reference genome (RefSeq assembly accession: GCF_003339765.1) using the Spliced Transcripts Alignment to a Reference (STAR) (57) package under default parameters. Quality mapped read counts were batch adjusted using the R package ComBat-Seq (58). Read counts were normalized to account for differences in sequencing depth among samples, filtered for genes with low counts or outliers, and differentially expressed genes were found using DESeq2 (59) under the apeglm (60) shrinkage estimator to reduce noise. Statistically significant differentially expressed genes between macaque groups were called at a significance α of 0.05 adjusted for multiple testing using the Benjamini-Hochberg FDR method and a fold change > 1.5. PCA was performed through the DESeq2 package to visualize the overall gene expression profile among individual macaques.

### Pathway analysis.

Pathway analysis of up- and downregulated DEGs in infected macaques were performed using both the clusterProfiler (61) R package and the ShinyGO (62) webapp to query the GO (63, 64) and KEGG (65) databases using overrepresentation analysis under a FDR cutoff < 0.05. Two query algorithms and 2 pathway databases were used to provide increased confidence in relevance of the identified pathway; only pathways shared between the 2 search algorithm results were retained. Functional pathways were visualized using the Pathview (66) R package. Additional pathway enrichment analysis was performed with GSEA 4.3.2. (67). Annotated gene sets from the Hallmark molecular signature database (68) and the blood transcriptome module (BTM) (69) were used for these analyses.

To assess potential differences in gene expression profile among infected macaques, these were initially stratified as progressors and controllers, as defined above. We assessed for differences in the overall gene expression profile using PCA before focusing on the expression of immune cell markers and cytokines/chemokines among these infected macaque groups. We also assessed the expression of genes that may be used as biomarkers of parasite control by identifying genes differentially expressed between controller and progressor macaques, whose protein products may be secreted and measured in blood/serum samples. LDA was performed to assess the ability of a set of biomarkers able to identify controller and progressor macaques. Significance of the LDA was assessed by 1-way PERMANOVA.

Next, we tested for further differentiation of host responses and performed an unsupervised hierarchical clustering of infected macaques based on a complete linkage analysis of their differential gene expression profiles, which calculates the maximum pairwise dissimilarity between elements in 2 clusters when building the clusters. The clustering was performed on a subset of 85 genes presenting a significant difference in expression level among infected macaques (*P* < 0.05, FDR-corrected; >1.5 fold change). Infected macaques were then stratified according to these new clusters to assess their immune and infection profiles. We also tested for the composition of parasite strains infecting macaques as a predictor for these new clusters, using LDA of parasite strain composition, and statistical significance was evaluated by 1-way PERMANOVA. The reclassification matrix was also calculated to estimate the accuracy of the LDA.

Multivariate analysis of the gene expression profile was also performed using the MADE4 package (70), which is specifically built to perform supervised dimension reduction and BGA using gene read count data. BGA is a supervised PCA method seeking to ordinate groups rather than individual samples (70).

### Statistics.

Changes in blood parasite burden were compared between progressor and controller macaques with 2-tailed Student *t* test. Differentially expressed genes were called at a significance α of 0.05 adjusted for multiple testing using the Benjamini-Hochberg FDR method and a fold change > 1.5 in DESeq2 (59) under the apeglm (60) shrinkage estimator to reduce noise. PCA was performed through the DESeq2 package. Pathways associated with differentially expressed genes were identified using overrepresentation analysis under a FDR cutoff < 0.05. BGA of differential gene expression was performed in MADE4 (70). LDA of parasite DTU composition and of biomarkers gene expression level was performed in PAST4 and statistical significance was assessed by 1-way PERMANOVA.

### Study approval.

The study was approved by the Tulane IACUC.

### Data availability.

All RNA-Seq has been deposited in NCBI SRA database and is available under BioProject PRJNA1139163 (Biosamples SAMN42764022–SAMN42764047) and BioProject PRJNA1010169 (Biosamples SAMN37182435, SAMN37182439, and SAMN37182443). Supporting Data Values are available in the supplement.

## Author contributions

ED designed the study and acquired funding with support from CH and PAM; HD contributed to the design of discrete experiments and analyses; HD, WT, and KG conducted the experiments and analyzed data with technical support and conceptual advice from ED and CH; and HD and ED prepared the manuscript, with review and editing input from all authors.

## Supplementary Material

Supplemental data

Supporting data values

## Figures and Tables

**Table 1:**
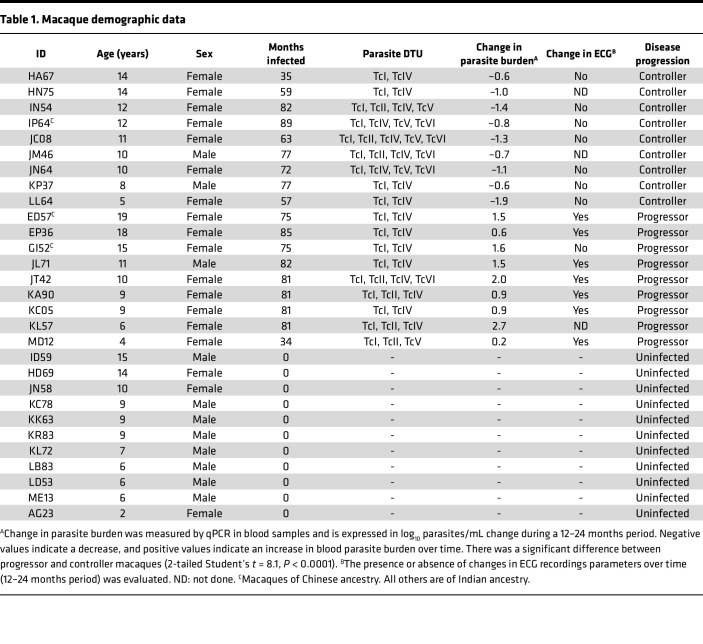
Macaque demographic data

## Version 1 01/07/2025

In-Press Preview

## Version 2 02/24/2025

Electronic publication
