# Supplementary material for: PBMC transcriptomic signatures reflect Trypanosoma cruzi strain diversity and trained immunity in chronically infected macaques
Source: JCI Insight. 2025 Jan 7;10(4):e186003. doi: 10.1172/jci.insight.186003 (PMC11949070; doi:10.1172/jci.insight.186003)
Supplement: Supplemental data [file jciinsight-10-186003-s079.pdf]

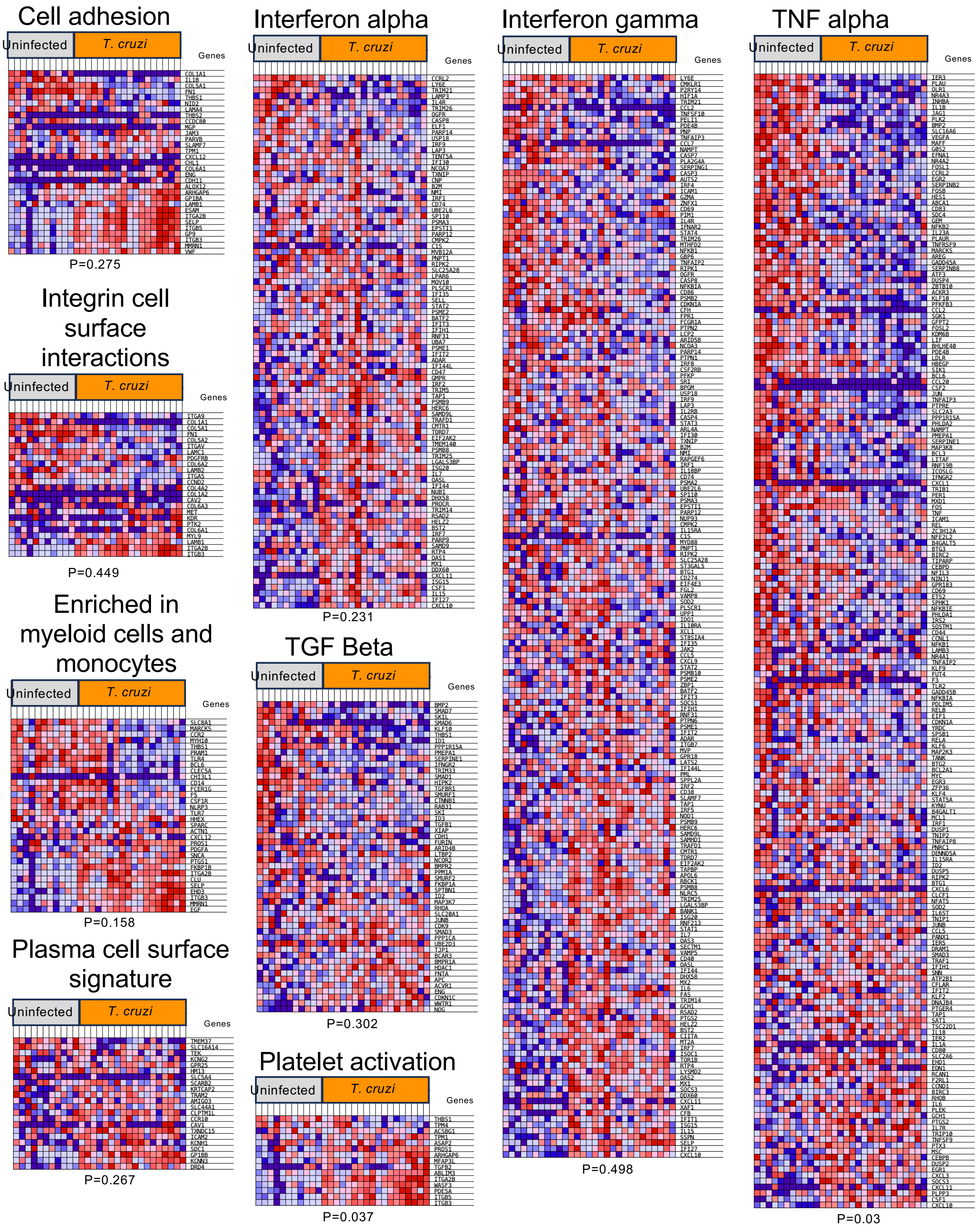


**Supplementary Figure 1. Enriched pathways in PBMCs from uninfected and *T. cruzi* infected macaques.**

GSEA was performed on the transcriptomic profile of PBMCs using the BTM and Hallmark modules, and heatmaps of gene expression for the indicated pathways are shown. The statistical significance of the enrichment of the respective pathways is indicated at the bottom of each heatmap.

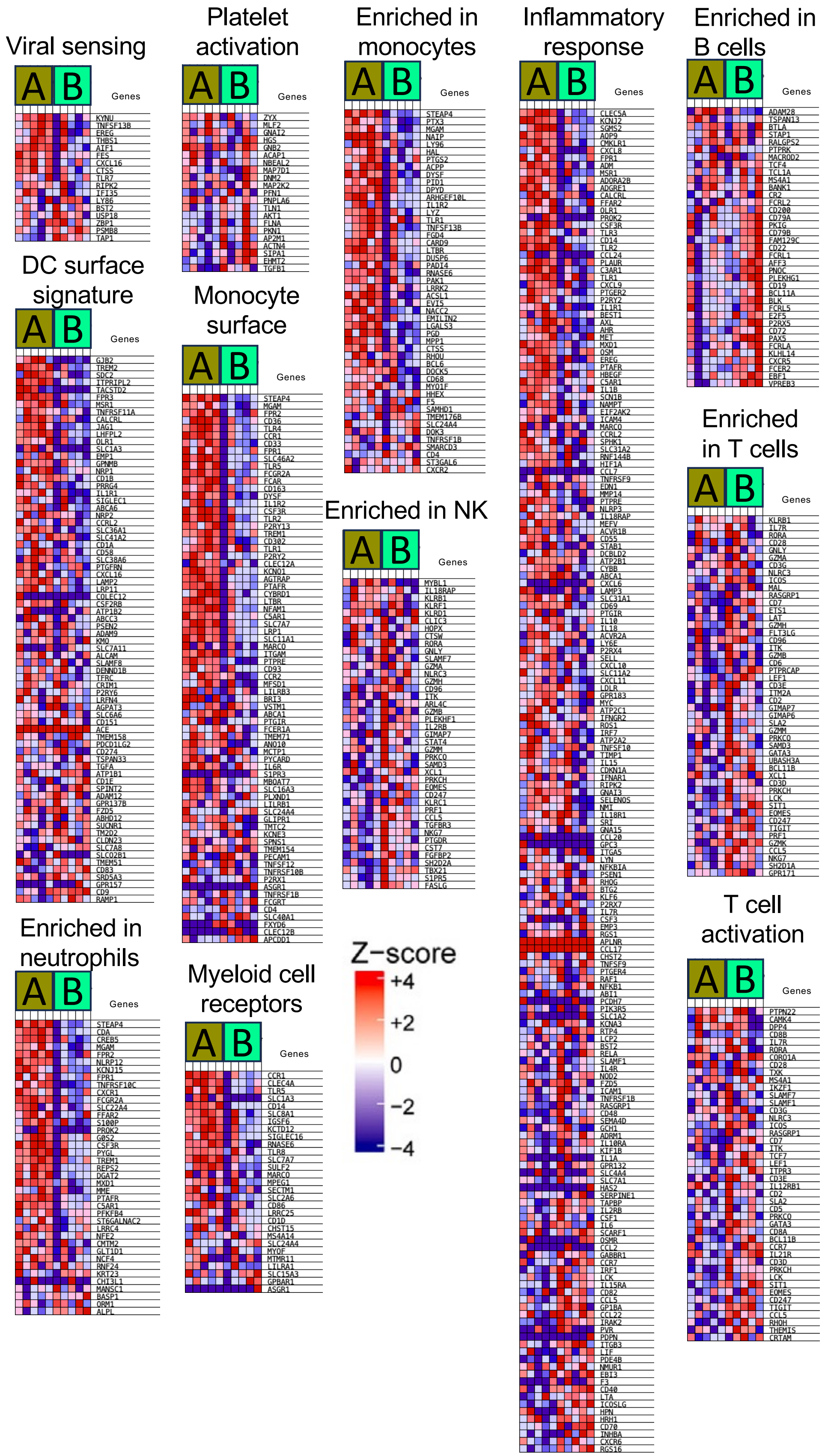


**Supplementary Figure 2. Enriched pathways in PBMCs from *T. cruzi* infected macaques from Clusters A and B.**

GSEA was performed on the transcriptomic profile of PBMCs using the BTM and Hallmark modules, and heatmaps of gene expression for the indicated pathways are shown.

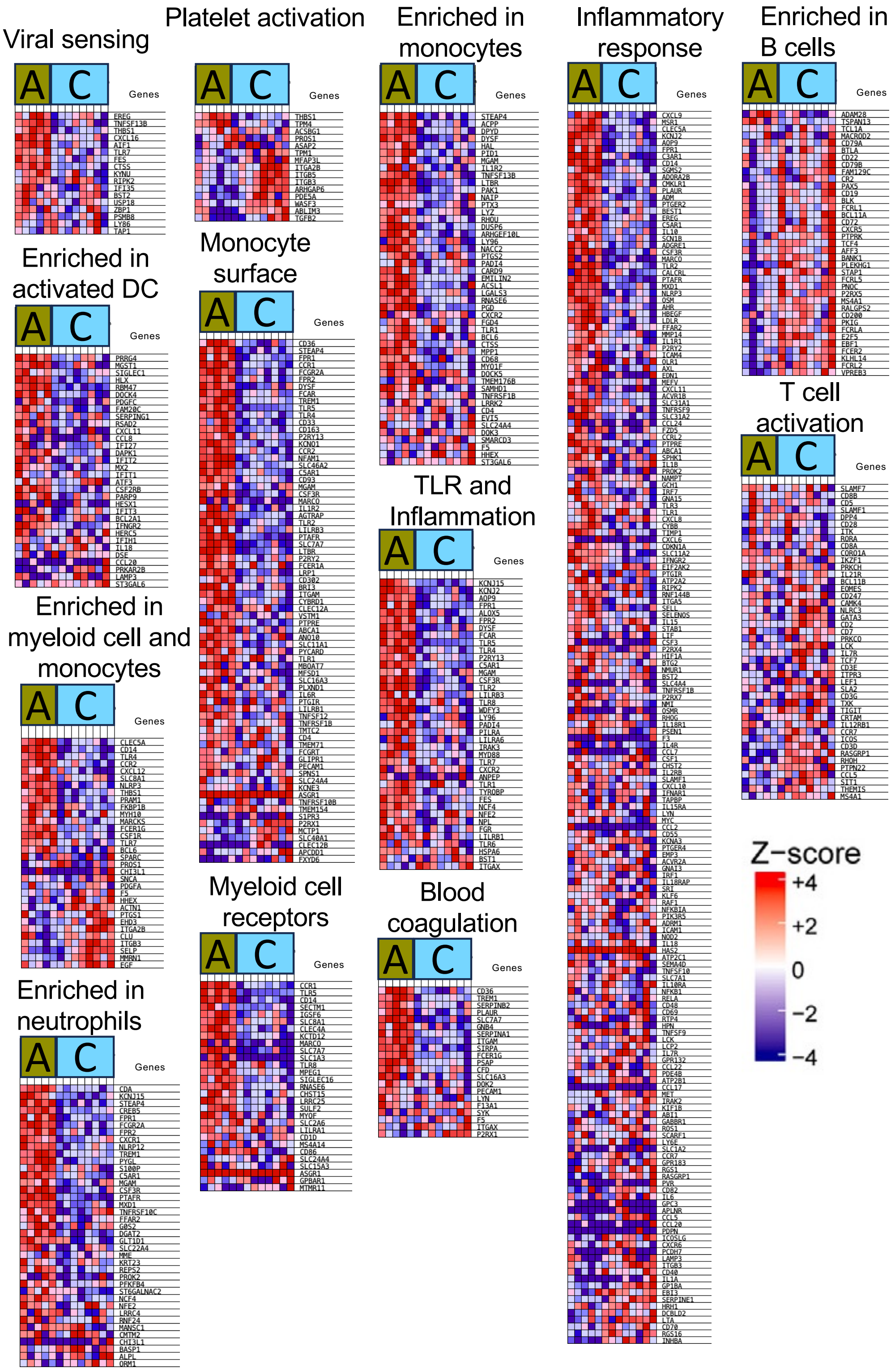


**Supplementary Figure 3. Enriched pathways in PBMCs from *T. cruzi* infected macaques from Clusters A and C.**

GSEA was performed on the transcriptomic profile of PBMCs using the BTM and Hallmark modules, and heatmaps of gene expression for the indicated pathways are shown.

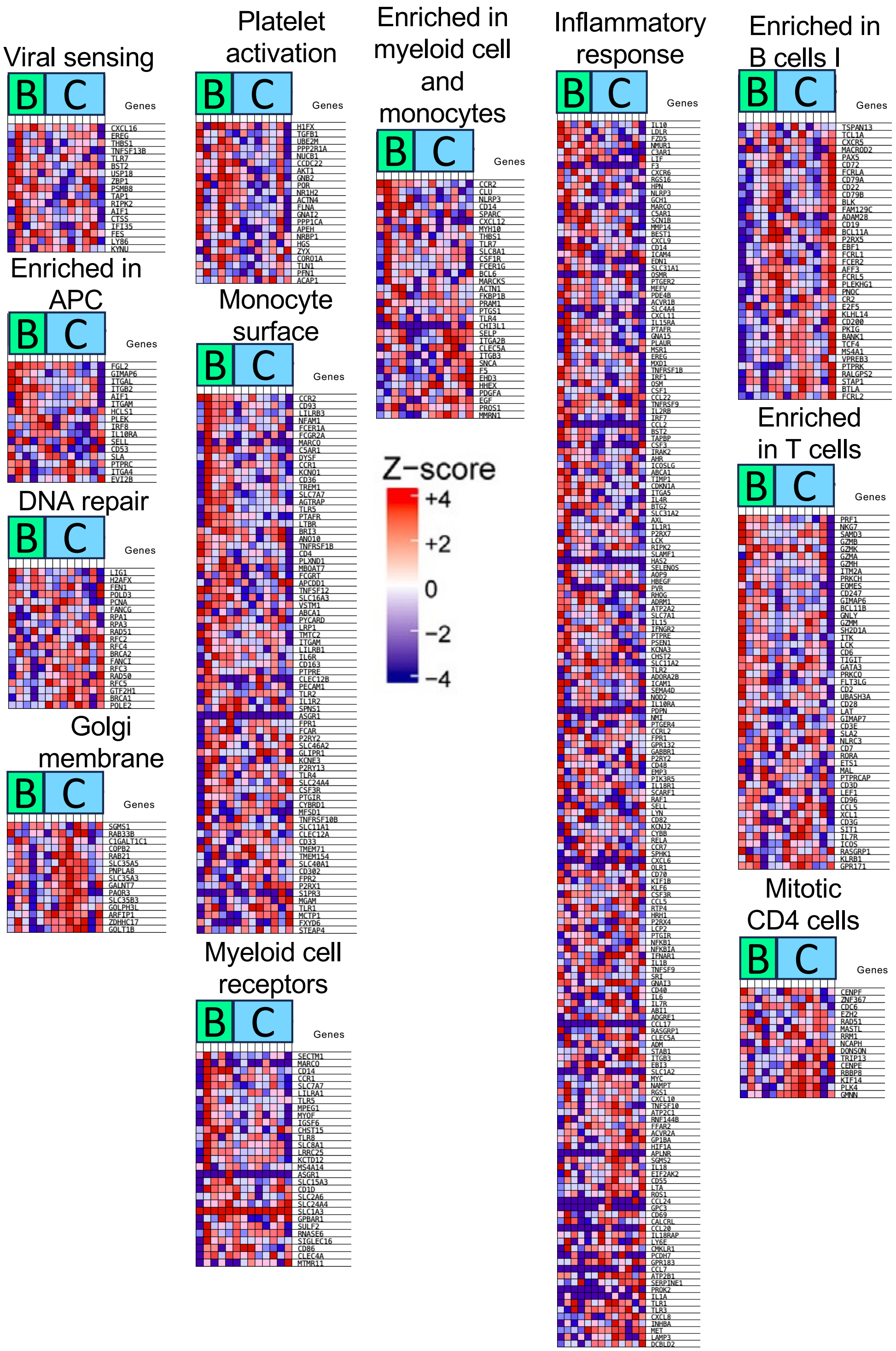


**Supplementary Figure 4. Enriched pathways in PBMCs from *T. cruzi* infected macaques from Clusters B and C.**

GSEA was performed on the transcriptomic profile of PBMCs using the BTM and Hallmark modules, and heatmaps of gene expression for the indicated pathways are shown.

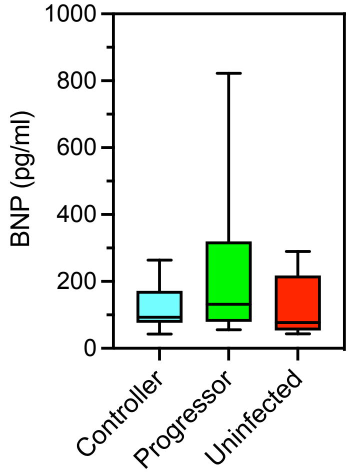


**Supplementary Figure 5. BNP plasma levels among macaques.**
Brain natriuretic peptide (BNP) concentration was measured in plasma samples from controller, progressor and uninfected macaques. Progressor macaque tended to have higher BNP levels, although this did not reach statistical significance (Kruskal-Wallis, P=0.22).
